# Using Machine Learning Methodology to Identify Predictors of Non-Response to MTSS-B: a Focus on Discipline Problems and Juvenile Justice Involvement

**DOI:** 10.1007/s11121-026-01932-0

**Published:** 2026-05-22

**Authors:** Kate Somerville, Ali Ünlü, Angela K. Henneberger, Bess A. Rose, Elise T. Pas, Catherine P. Bradshaw

**Affiliations:** 1 University of Virginia School of Education and Human Development, Charlottesville, VA USA; 2 University of Maryland School of Social Work, Baltimore, MD USA; 3 Johns Hopkins Bloomberg School of Public Health, Baltimore, MD USA

**Keywords:** Tiered intervention, School discipline, Justice involvement, School-based prevention, Predictive modeling, Machine learning

## Abstract

Multi-tiered systems of support for behavior (MTSS-B) is a widely-used tiered preventive intervention currently used in over 25,000 schools across the USA. This study leveraged data from 16,907 elementary students across 42 schools that participated in a prior randomized controlled trial (RCT) of MTSS-B, where schools were randomly assigned to implement Tier 1 + 2 (intervention) supports or Tier 1 only (comparison). The RCT data, collected between 2008 and 2012, were linked to administrative records of behavior in grades 6–12 collected between 2008 and 2024. We used machine learning methods to identify elementary school predictors of three long-term outcomes (i.e., in-school suspension (ISS), out-of-school suspension (OSS), and arrest) signifying Tier 1 non-response. For ISS, the strongest predictors were family involvement, being female, and internalizing problems, which serve as protective factors. For OSS, they were family involvement, internalizing problems, and academic performance, all of which were protective. For arrest, key predictors included family problems, where lower problems were protective against arrest, and prosocial behavior and family involvement, which were both protective. The top predictors were similar for students in the Tier 1 + 2 and Tier 1 only conditions. These findings can inform early screening for possible non-response to MTSS-B and highlight the need for family engagement practices.

**Supplementary Information:**

The online version contains supplementary material available at https://doi.org/10.1007/s11121-026-01932-0.

Students who display disruptive or aggressive behavior in elementary school face elevated risk for special education referral, school failure, and criminal justice involvement (National Academies of Sciences, Engineering, and Medicine, 2019). Preventive behavioral intervention frameworks like multi-tiered systems of support for behavior (MTSS-B; Sugai & Horner, 2020) offer an alternative to exclusionary discipline (e.g., suspension, expulsion), ostensibly reducing the number of students pushed into the school-to-prison pipeline (Mitchell & Bradshaw, 2013). Drawing on a public health model (O’Connell et al., 2009), the MTSS-B framework asserts that universal (Tier 1) supports effectively promote positive behavior for all students, while targeted (Tier 2) and intensive (Tier 3) supports are needed for students not responding to the Tier 1 supports (Sugai & Horner, 2002).

MTSS-B has shown positive effects on behavioral outcomes, including teacher-rated behavioral and mental health problems and bullying (Bradshaw et al., 2010a, 2010b). However, the model is built on the premise that some students will not respond to lower-tier interventions (e.g., a universal intervention at Tier 1) and will require more intensive support and intervention to reduce behavioral risk (Bradshaw et al., 2012a, 2012b). Yet relatively little is known about how to predict which students are most likely to need advanced tier supports. Using a predictive modeling approach, the current paper identifies factors associated with discipline outcomes among students attending schools with tiered interventions, within a study comparing Tier 1 vs. Tier 1 + 2 preventive interventions. Early identification of likely non-responders allows for timely intervention and may prevent a cascade of negative outcomes (Masten & Cicchetti, 2010). We draw on administrative and follow-up data from a randomized trial of MTSS-B to examine in-school suspension, out-of-school suspension, and arrest to understand early predictors of long-term non-response to the preventive intervention.

## MTSS-B as a Tiered Framework for Preventing Behavior Problems

From a life course perspective (Catalano & Hawkins, 1996; Elder, 1998), early academic and behavioral experiences carry strong implications for later outcomes (Entwisle et al., 2003; Masten & Cicchetti, 2010; Patterson et al., 2010). Early disruptive behavior often leads to removal from academic settings through time out, suspension, or special education placement limiting students’ exposure to positive educational and social experiences (Reid, 1993). This cycle of exclusion and reinforcement is a key contributor to the school-to-prison pipeline, wherein students removed from school are at greater risk for arrest (Christle et al., 2005), particularly racially minoritized students and students with disabilities (Lerner & Galambos, 1998; Skiba et al., 2002; Wald & Losen, 2003).

MTSS-B is a tiered prevention framework through which schools organize evidence-based behavioral interventions. Positive Behavioral Interventions and Supports (PBIS) is one example of such a framework. These tiered support frameworks aim to establish consistent positive behavioral expectations across all school settings and equip staff with effective strategies for responding to student concerns. These changes are theorized to reduce behavioral problems and minimize disruptions for students and teachers. Implementation is sustained through data-based decision making and systemic supports (e.g., teaming, coaching, technical assistance), with the ultimate goal of reducing behavioral problems and increasing academic opportunity for all students (for a review, see Horner et al., 2025).

A growing body of research demonstrates the positive impacts of the Tier 1 model (see Lee & Gage, 2020 for a review), including significant effects on student behavior, social emotional, and academic outcomes and the reduction in student need for additional supports (e.g., Bradshaw et al., 2009, 2010a, 2010b, 2012a, 2012b; Horner et al., 2009, 2025). A quasi-experiment of the effectiveness of Tier 1 scale-up in Maryland utilized propensity score weighting and demonstrated significant impacts on academics and reduced suspensions (Pas et al., 2019). Similarly, a Florida-based study found that schools implementing PBIS Tier 1 with fidelity demonstrated lower suspension rates than matched non-PBIS schools (Gage et al., 2019).

Consistent with the MTSS-B framework, not all students’ behavioral needs are met by Tier 1 supports, necessitating Tier 2 targeted interventions (Sugai & Horner, 2002; Weist et al., 2024). Unlike the individualized approach of Tier 3, Tier 2 supports are typically delivered to groups of students with similar needs and require relatively little time and resources to implement. Widely used examples include Check-In/Check-Out and function-based supports (for a review, see McIntosh et al., 2009). Research demonstrates that Tier 2 interventions significantly reduce office discipline referrals (Drevon et al., 2019; Hawken et al., 2014; Mitchell et al., 2011; Todd et al., 2008) and increase academic engagement (Drevon et al., 2019). An RCT comparing Tiers 1 + 2 to Tier 1 only found significant effects on teacher efficacy, special education service use, and academic performance (Bradshaw et al., 2012a, 2012b).

## Identifying Predictors of Non-response to MTSS-B: Variation in Responsiveness to Prevention Programming

The effects of universal prevention programs may not be consistent across all exposed students (Bradshaw et al., 2015), and baseline risk has been a particular focus of research on variation in response to universal interventions. Bradshaw et al. (2015) found that Tier 1 MTSS-B effects were stronger for students whose baseline behaviors indicated “at-risk” or “high-risk” status compared to students whose baseline behaviors suggested they were average or socially skilled. Similarly, the Good Behavior Game effectively reduced the likelihood of antisocial personality disorders, harmful health behaviors, and externalizing and internalizing behaviors among students with elevated baseline disruptive and aggressive behavior, emotional problems, or victimization experiences (Kellam et al., 2008; Spilt et al., 2013), though students with family problems or other combinations of social and behavioral risks showed fewer benefits (Spilt et al., 2013).

Relatively few studies have examined variation in response to Tier 2 interventions, and those that have focused primarily on behavioral function. McIntosh et al. (2009) found that Tier 2 effectiveness varied by behavioral function. Interventions were effective for students with attention-maintained behavior but not for those with escape-maintained behavior, a pattern also found in subsequent research (Carter & Horner, 2007; Eklund et al., 2019).

Within a response to intervention framework (Hawken et al., 2008), persistent behavioral problems in the presence of a preventive intervention signal non-response and the need for a higher tier of support. Yet many schools struggle to meet the needs of Tier 1 non-responders (Pas et al., 2019). Greater insight into students who continue to experience behavior and discipline problems after exposure to Tier 1 + 2 supports can inform the tailoring of Tier 3 interventions and modification of Tier 2 supports when appropriate (Lane, 2007). However, no current studies have examined predictors of non-response comparing students exposed to Tier 1 alone versus Tier 1 + 2, a gap with important implications for developing screening tools to guide referral to higher-tier interventions.

Individual demographic characteristics also predict suspension and expulsion, reflecting structural factors such as institutional racism, discrimination, and bias that disadvantage students with particular social identities (Skiba & Rausch, 2013). Racially minoritized students, students with disabilities, and males are disproportionately subject to exclusionary discipline (Leung-Gagné et al., 2022). While we remain largely focused on more malleable risk factors of non-response, these demographics are included as covariates in our predictive modeling.

## The Current Study

Understanding early predictors of non-response is critical for screening and tailoring supports across MTSS-B tiers (Bradshaw et al., 2015; Lane, 2007). Using machine learning (ML), the current study identified student characteristics, teacher-rated variables, and behavioral data most predictive of three outcomes indicating non-response measured using administrative data from the Maryland Longitudinal Data System (MLDS) in grades 6–12: in-school suspension (ISS), out-of-school suspension (OSS), and juvenile arrest (aim 1). Data were linked from a prior RCT in 42 elementary schools comparing Tier 1 + 2 versus Tier 1 only, enabling examination of early predictors of adolescent non-response, as measured by exclusionary discipline and arrests, up to 12 years after Tier 1 or Tier 1 + 2 supports were delivered. Although malleable predictors were the primary focus, race, sex, and free-reduced meals status were included as covariates given their established associations with behavior problems and justice involvement (Scott et al., 2017).

For aim two, we compared the relative strength of predictors across intervention and comparison conditions using ML and complemented by hierarchical linear modeling (HLM). Generating separate ML algorithms for each condition allowed us to examine how access to Tier 2 supports may shift associations between predictors and non-response. For example, if externalizing behavior emerged as the top risk factor in the comparison group but ranked 5th or 6th in the intervention group, this would suggest Tier 1 + 2 access disrupted its association with non-response in a way Tier 1 alone did not. Importantly, while the RCT design allows comparison of predictor-outcome associations across conditions, it does not consider whether the intervention itself caused reduced non-response. Rather, our approach draws on concepts of developmental cascades (Masten & Cicchetti, 2010) and variation in responsiveness to preventive interventions (Bradshaw et al., 2015) to understand non-response risk using ML methodology.

## Method

### Study Context

Data come from the “PBISplus” RCT, which tested Tier 1 + 2 supports versus Tier 1 only across 42 elementary schools recruited through a research-practice partnership among university researchers, the state, and participating school divisions (see Bradshaw et al., 2012a, 2012b, for additional details). All eligible schools were those in participating districts that had already received training in Tier 1 MTSS-B/positive behavior support and reached high-fidelity Tier 1 MTSS-B implementation but had an expressed need for Tier 2 supports, evidenced by a sufficient number of Tier 1 non-responders. Using a group RCT design (Murray, 1998), schools were stratified by district, matched on baseline suspensions and select school demographics (e.g., percent of students receiving free/reduced meals, school size), and randomized to Tier 1 + 2 (*n* = 20) or Tier 1 only (*n* = 22), with the latter continuing universal supports with “support as usual” from the district and state.

Schools in the Tier 1 + 2 condition received external training and coaching, paid for and provided by the research team, supporting implementation of Tier 2 interventions (e.g., Check-In/Check-Out) and evidence-based practices (e.g., function-based interventions) at both the team and individual teacher level. Findings from the original 3-year RCT demonstrated improvements in special education service use, student academic performance, and teachers’ behavioral management efficacy in the Tier 1 + 2 condition relative to Tier 1 only (Bradshaw et al., 2012a, 2012b).

### Sample

A total of 16,907 students had baseline data collected in one of the 42 elementary schools participating in the initial RCT (see Bradshaw et al., 2012a, 2012b, for details on the initial RCT) and could be linked to the outcomes (see CONSORT in Figure S1). Baseline measures were collected prior to intervention implementation across two cohorts beginning in summers 2007 and 2008. These data were linked to suspension and arrest records from the MLDS (2008–2024). Descriptive statistics and race/ethnicity information are provided in Tables 1 and S1, respectively.

**Table 1:** Variable definitions and descriptive statistics. (*N* = 16,907)

Variable	Definition	*N* (%)	M (SD)
Free/reduced price lunch	Does this student qualify for free or reduced price lunch?	6991 (41.3%)	
Female	Does this student identify as male (0) or female (1)?	8032 (47.5%)	
Minority	Nonwhite racial/ethnic identity	11,180 (66.1%)	
Grade level K	Student started the study in Kindergarten	2688 (15.8%)	
Grade level 1	Student started the study in Grade 1	2670 (15.8%)	
Grade level 2	Student started the study in Grade 2	2725 (16.1%)	
Grade level 3	Student started the study in Grade 3	2899 (17.1%)	
Grade level 4	Student started the study in Grade 4	2898 (17.1%)	
Grade level 5	Student started the study in Grade 5	2893 (17.1%)	
Teacher reports of academic performance, behavior, family involvement, and school-based services			
Academic performance	Baseline teacher assessment of student academic performance		3.16 (1.20)
Concentration problem	Baseline teacher assessment of student concentration problems		2.69 (1.18)
Disruptive behavior	Baseline teacher assessment of student disruptive behavior		1.83 (0.78)
Emotion problem	Baseline teacher assessment of student emotional problems		2.20 (1.03)
Internalizing problem	Baseline teacher assessment of student internalizing problems		1.83 (0.79)
Prosocial behavior	Baseline teacher assessment of student prosocial behavior		4.78 (0.98)
Family involvement	Baseline teacher assessment of student family involvement		4.56 (1.30)
Family problem	Baseline teacher assessment of student family problems		3.06 (0.59)
Academic services	Does this student receive academic services?	3388 (20%)	
Behavioral services	Does this student receive behavioral services?	1203 (7.1%)	
English language learner	Does this student qualify for ELL services?	778 (4.6%)	
Principal’s office	Baseline number of visits to the principal’s office	822 (4.9%)	
Psychological services	Does this student receive psychological services?	862 (5.1%)	
Special education	Does this student receive special education services?	2110 (12.4%)	
Special education assessment	Has this student been referred for a special education assessment?	800 (4.7%)	
Student support team referral	Has this student been referred to the student support team?	1225 (7.2%)	
Outcomes: suspension and juvenile justice involvement			
In-school suspension	Student experienced ≥ 1 in-school suspension between grades 6 and 12	1981 (11.7%)	
Out-of-school suspension	Student experienced ≥ 1 out-of-school suspension between grades 6 and 12	4785 (28.3%)	
Arrest	Student experienced ≥ 1 arrest between grades 6 and 12	1884 (11.1%)	

Baseline measures were collected when students were in kindergarten through 5th grade (Cohort 1: 2008; Cohort 2: 2009). Outcomes reflect any ISS, OSS, or arrest during grades 6–12

The machine learning analysis used a binary Minority variable (0 = White, 1 = all other categories) rather than specific racial/ethnic categories, as the analytic approach required binary predictors and disaggregating race/ethnicity threatened to dissolve its predictive power. This operationalization reflects scholarship on how systemic racism positions whiteness as the assumed norm in USA schooling. We acknowledge that collapsing minoritized students into a single category inadequately captures their diverse and intersecting identities. The full racial/ethnic breakdown is presented in Supplemental Table S1

### Measures

Predictor variables collected during the RCT included baseline demographic characteristics, teacher-reported academic performance, behavior, service use, and family involvement, supplemented by demographic data from the MLDS (see Table 1 for full descriptions). Teacher-reported academic performance and behavior were measured using the *Teacher Observation of Classroom Adaptation* checklist (TOCA-C; Bradshaw & Kush, 2020; Bradshaw et al., 2010a, 2010b), based on the TOCA-R (Koth et al., 2009). Teachers rated each student on a 6-point Likert scale (1 = “never” to 6 = “almost always”) across seven subscales: concentration problems (*α* =.93), internalizing problems (*α* =.86), aggressive/disruptive behavior (*α* =.87), prosocial behavior (*α* =.84), emotion regulation problems (*α* =.87), family problems (*α* =.82), and family involvement (*α* =.93). Item response theory, item difficulty, and confirmatory factor analyses indicated limited bias by gender, race, or grade (see Bradshaw and Kush [2020] for additional psychometric details). Teachers also indicated whether students had received the referrals or school-based experiences listed in Table 1 and rated academic performance on a 5-point scale (poor to excellent) at baseline (mid-to-late September). Condition (Tier 1 only vs. Tier 1 + 2) and cohort (Cohort 1 vs. Cohort 2) variables were also included.

Three non-response outcomes were drawn from the MLDS, a statewide data system linking records across Maryland agencies. In-school suspension (ISS) and out-of-school suspension (OSS) data came from the Maryland State Department of Education, and arrest data from the Maryland Department of Juvenile Services. Each outcome was dichotomized to indicate whether the student ever experienced the event between 6 and 12th grades. Descriptive statistics for all study variables are reported in Table 1.

### Procedure

Homeroom teachers provided baseline TOCA-C ratings for all K–5 students in their classrooms during the RCT (see Bradshaw et al., 2012a, 2012b), using an open cohort design in which all students and staff who entered participating schools were eligible for inclusion. Data were collected in fall 2007 or 2008 for 90.4% of eligible children. The study was approved by the Institutional Review Board with a waiver of active parental consent.

### Data Analysis Rationale

ML was selected over traditional techniques (e.g., regression, latent class analysis) for several reasons. Its computing power can accommodate a larger number and wider range of variables, particularly valuable for predicting a complex outcome like non-response. ML also supports data-driven theory development analogous to grounded theory in qualitative research (Glaser & Strauss, 2017), strengthening our understanding of persistent discipline and behavior problems in schools providing tiered supports. Finally, ML can detect non-linear and higher-order predictor-outcome associations (Leist et al., 2022) beyond the linear models traditionally used in social science. We supplement this approach with HLM to account for student nesting within schools and compare the relative strength of key predictors.

### Data Pre-Processing

Predictor missingness ranged from 0 to 10%, with the highest rates for teacher-reported psychology services (10%; *n* = 1731); family problems (3.6%; *n* = 617); family involvement (2.8%; *n* = 478); and academic services (2.5%; *n* = 418). All remaining predictor variables had minimal missingness. Outcome missingness (8%; *n* = 1471) occurred when students relocated out of state prior to sixth grade and exited the state data system.

Missing data were largely balanced across intervention and comparison groups and cohorts. Missingness on most demographic and behavioral indicators (e.g., minority status, gender, and family problems) typically varied by less than 1%. The largest observed difference in missingness occurred in family involvement (1.49% higher in the comparison group) and psychological services (1.03% higher in the comparison group), suggesting that data attrition or non-response bias was balanced across conditions.

Missing data were addressed using multiple imputation by chained equations (MICE) in R (version 4.5.1), which yields valid estimates under the Missing at Random (MAR) assumption in nested data. Five imputed datasets were generated using a multilevel imputation approach accounting for student nesting within schools, with school ID specified as the cluster identifier. All study variables were retained in the imputation model including those not analyzed in the current study (e.g., reading score, math score, county identifiers), to preserve its integrity. Pairwise Pearson correlations revealed two notably high correlations: disruptive behavior with emotional problems (*r* =.75, *p* <.001) and with prosocial behavior (*r* =  −.71, *p* <.001). All other pairwise correlations fell below this threshold.

### Prediction Models

We followed a consistent analytical framework across all modeling stages, including data standardization (RobustScaler; scikit-learn), tenfold cross-validation, and SMOTE-Tomek to address class imbalance. Ten-fold cross-validation partitions data into ten subsets, iteratively training on nine and validating on one, reducing overfitting and providing reliable performance estimates (Pedregosa et al., 2011). SMOTE-Tomek is a hybrid preprocessing technique that oversamples the minority class (here, occurrence of ISS, OSS, or arrest) by generating synthetic samples based on existing feature space (Li et al., 2022), while removing ambiguous samples near decision boundaries (Tomek, 1976). This improves class separability, or the degree to which observations from different outcome categories are distinct and distinguishable in the feature space and supports more robust model training and evaluation (Pedregosa et al., 2011). Results without SMOTE served as a sensitivity analysis, confirming improved predictive performance without distorting underlying data distributions (Fernández et al., 2018; Unlu et al., 2025).

Twelve supervised learning algorithms were evaluated using default hyperparameters to identify the best-fitting model for each outcome. Model performance was compared using F1-scores, or the harmonic mean of precision and recall, which is a particularly appropriate metric when outcome classes remain imbalanced even after resampling. Given evidence that base ML models often outperform deep learning on tabular data (Unlu & Subasi, 2025; Unlu et al., 2023), only one deep learning model (artificial neural networks (ANNs)) was included. Algorithms tested were: Logistic Regression, Linear Discriminant Analysis, k-Nearest Neighbors, Decision Trees, Naïve Bayes, AdaBoost, Gradient Boosting Machines, Random Forest, Extra Trees, Bagging, XGBoost, and ANNs (Pedregosa et al., 2011).

The top four models underwent hyperparameter tuning via RandomizedSearchCV (Pedregosa et al., 2011), which searches predefined hyperparameter distributions to identify the optimal configuration for each algorithm. Tuned models were evaluated on accuracy, precision, recall, F1-score, Cohen’s Kappa, Matthews Correlation Coefficient (MCC), and the Area Under the Receiver Operating Characteristic Curve (ROC-AUC; Raschka et al., 2022), with the best-performing model selected for interpretability analysis. Values above 0.7 are generally considered indicative of adequate to strong predictive performance, values above 0.5 indicate performance above random chance, and AUC-ROC above 0.90 suggests excellent discrimination. However, these thresholds are not universally fixed and performance metrics are influenced by data quality, model architecture, and ML methodology (Raschka et al., 2022).

### Shapley Additive Explanations

To interpret prediction model results, we employed SHAP (SHapley Additive exPlanations), which quantifies individual feature contributions to model predictions at both the dataset and individual-level by measuring the extent to which each predictor increases or decreases the probability of a given outcome (Lundberg & Lee, 2017). SHAP was applied to the best-performing model for each outcome to identify the top 10 most influential predictors, assess overlapping patterns of importance, and evaluate how each feature differentiated students with outcome records (i.e., suspension and arrest; class = 1) and those without outcome records (class = 0; Lundberg et al., 2020). Mean absolute SHAP values were then compared across conditions to examine whether predictor importance or ranking differed by intervention condition.

### Hierarchical Linear Modeling

A multilevel generalized linear mixed modeling approach for binary outcomes was implemented using the glmer function in the lme4 package in R (Bates et al., 2015), with students (level 1) nested within baseline schools (level 2). Null models were run to estimate between-school variance for each outcome, assuming a logistic distribution with level 1 variance of π^2^/3 ≈ 3.29 (Hox et al., 2018). The top 10 SHAP-identified features were then entered as predictors to assess the relative strength and direction of each. Models were first run for the full sample with TOCA-C scales grand-mean centered, then separately for each condition with scales centered within each sample (Raudenbush & Bryk, 2002).

## Results

### Prediction Models

Model screening across outcomes identified several top performing ensemble learning algorithms: Gradient Boosting Classifier (GBC), which builds models sequentially to correct previous errors; Extreme Gradient Boosting (XGBoost), an optimized version of gradient boosting; Extra Trees (Extremely Randomized Trees); Bagging (Bootstrap Aggregating); and Random Forest. These models were selected for hyperparameter tuning. Among them, Random Forest, an ensemble method that operates by constructing a multitude of decision trees, consistently achieved the highest predictive performance across all outcomes.

Although XGBoost performed comparably for arrest and Bagging for ISS, Random Forest was selected for its overall stability and consistency across outcomes. Algorithm performance varied more substantially without SMOTE. For each outcome, SMOTE and non-SMOTE models were compared to evaluate the impact of class imbalance correction; primary results are reported in Table 2, with non-SMOTE results in Table S2.

**Table 2:** Results for the top performing machine learning algorithm predicting suspensions and arrests

Outcome	Model	Accuracy	Precision	Recall	F1 score	Cohen Kappa	MCC	ROC AUC
Arrest								
	Random Forest	0.87	0.87	0.87	0.87	0.75	0.75	0.87
In school suspension								
	Random Forest	0.92	0.92	0.92	0.92	0.84	0.84	0.92
Out of school suspension								
	Random Forest	0.83	0.83	0.83	0.83	0.65	0.65	0.83

*MCC* is Matthews correlation coefficient, *ROC AUC* is the area under the receiver operating characteristic curve; outcomes are measured using a dichotomous indicator for whether the student experienced the event between 6 and 12th grade

The arrest prediction model demonstrated balanced performance, with accuracy, precision, recall, F1, and ROC-AUC all reaching 0.87, and Cohen’s Kappa and MCC of 0.75 (Table 2). The ISS model showed stronger predictive capacity, achieving 0.92 across all key metrics and agreement measures of 0.84 for both Kappa and MCC (Table 2). The OSS model performed somewhat lower, with 0.83 accuracy and consistent precision, recall, F1, and ROC-AUC scores, and Kappa and MCC of 0.65—above the 0.40 threshold indicative of moderate to substantial agreement (Landis & Koch, 1977), though below the other two models (Table 2).

SMOTE improved classification balance and model stability by generating synthetic minority class samples, reducing majority class bias and enabling more representative decision boundaries. The ISS model yielded the highest predictive scores, partly attributable to SMOTE generating a greater volume of synthetic data due to its higher class imbalance. Without SMOTE, weighted F1 scores were 0.88 for ISS, 0.75 for OSS, and 0.75 for arrest (Table S2), with notably lower ROC-AUC scores indicating reduced discriminatory power. The lower non-SMOTE performance reflects both the absence of synthetic samples and a trade-off of maintaining a standardized F1-based tuning pipeline which is optimized for balanced distributions rather than employing imbalance-specific optimization strategies, resulting in systematic misclassification toward the majority class. Overall, SMOTE yielded approximately 8–10% improvement in accuracy across all models and was therefore used for all reported results.

**Fig. 1:**
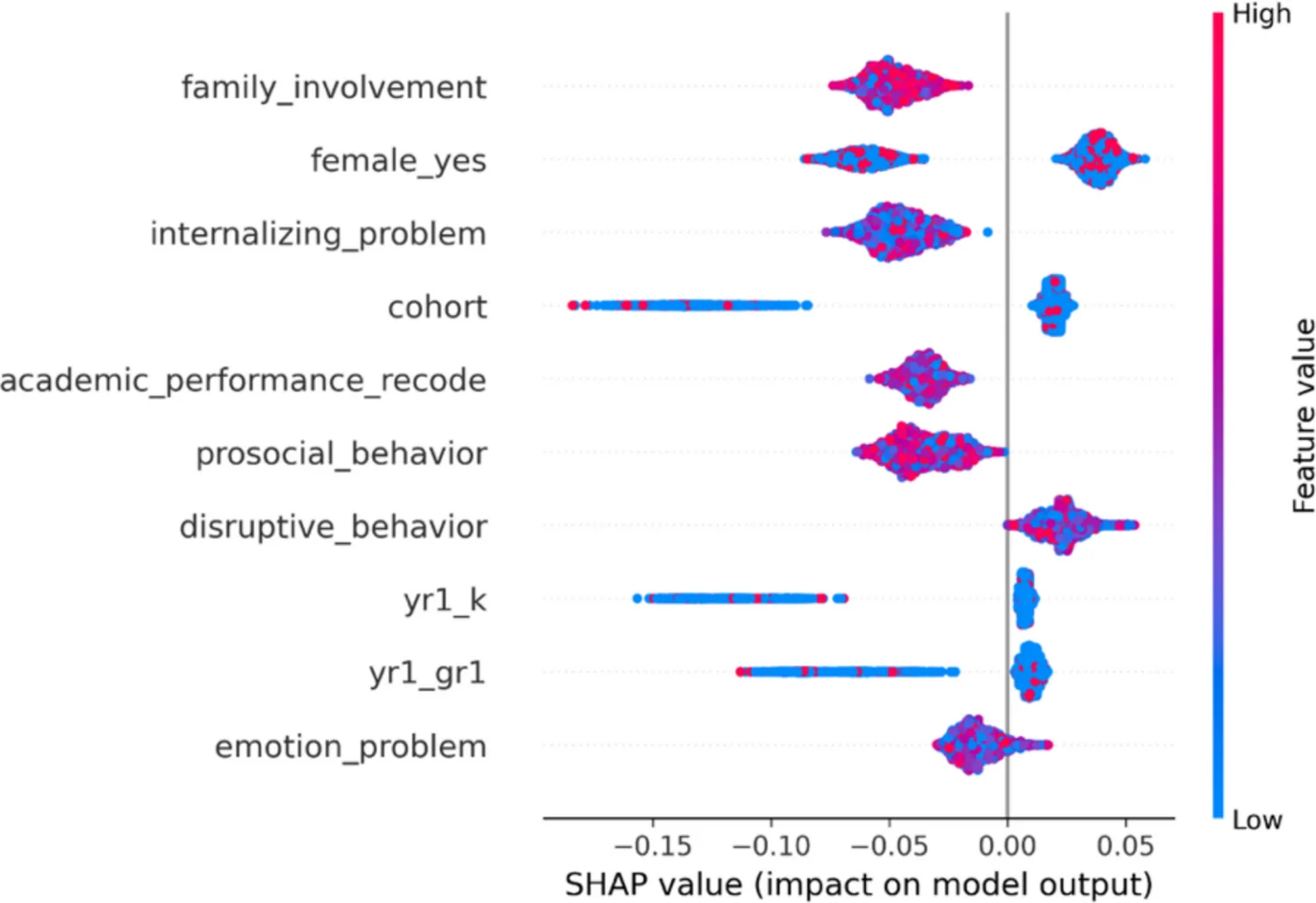
ISS beeswarm native plot

**Fig. 2:**
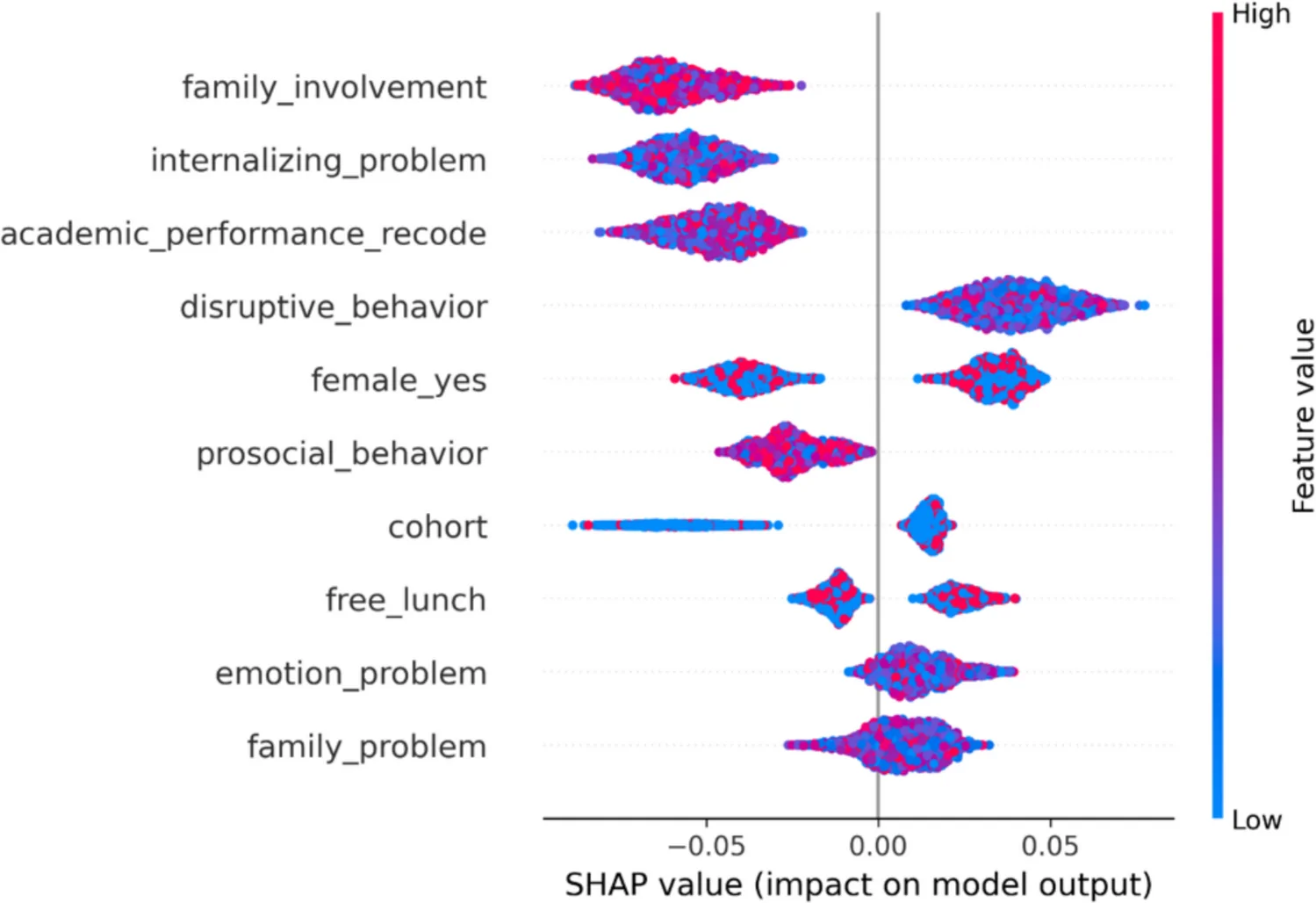
OSS beeswarm native plot

**Fig. 3:**
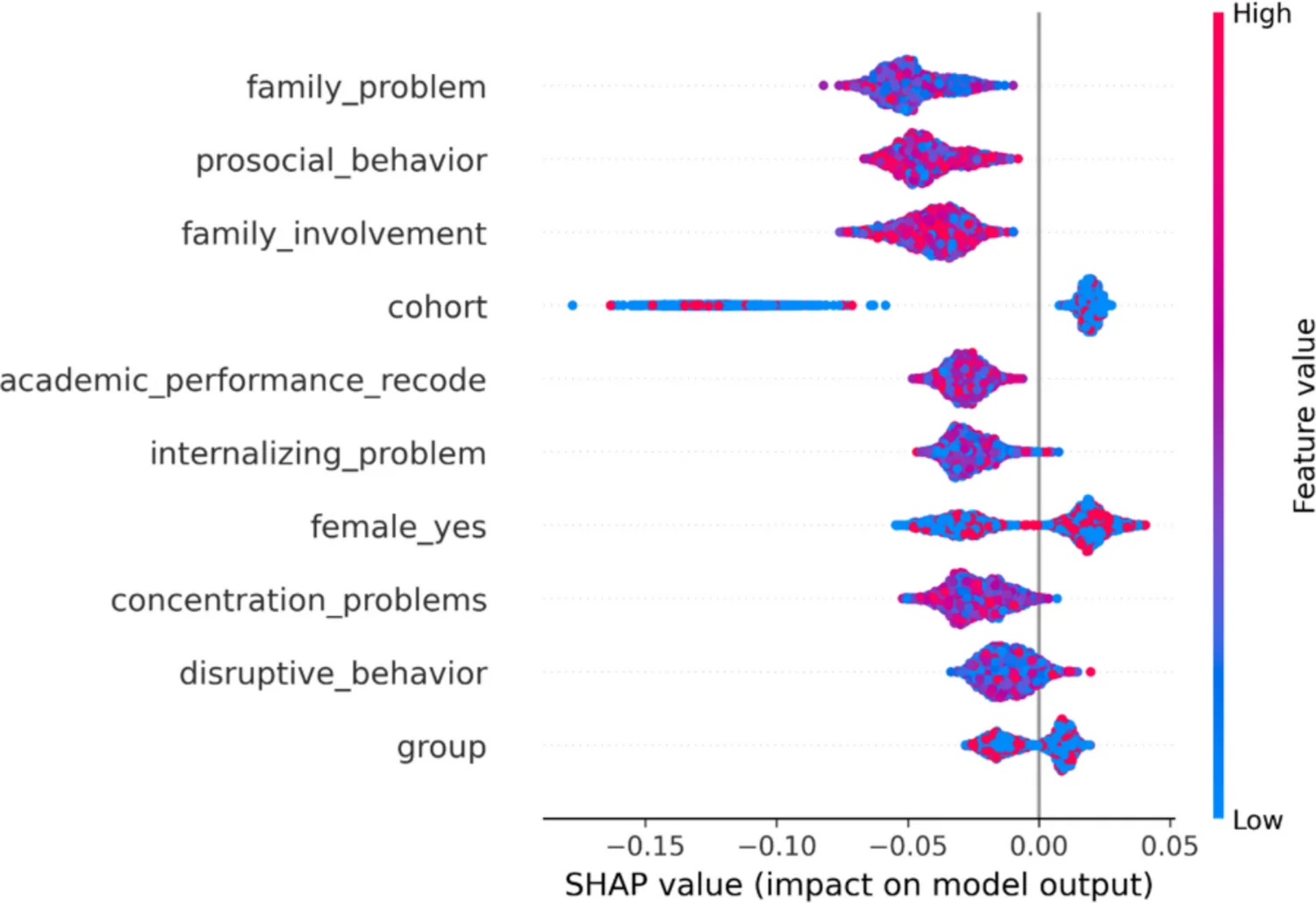
Arrest beeswarm native plot

Beeswarm plots (Lundberg & Lee, 2017) were used to visualize the strength and direction of each predictor’s influence across all observations in the SHAP analysis (Figs. 1, 2, 3). Each dot represents one case, positioned along the *X*-axis according to its SHAP value, or the degree to which that predictor shifts the predicted probability of the outcome. Dots to the right indicate increased probability and dots to the left indicate decreased probability. Color reflects the original feature value (red = higher, blue = lower), allowing interpretation of whether high or low feature values drive the effect. The distribution of dots reveals whether the effect is consistent across individuals or varies depending on feature value and context.

### Top Predictors of In School Suspension 

Figure 1 shows the top 10 predictors of ISS. *Family involvement* emerged as the strongest protective factor, with higher values associated with negative SHAP values indicating a protective effect; greater family involvement reduced ISS likelihood. A similar protective pattern was observed for gender, with *female* students showing decreased predicted probability of ISS. *Internalizing problems* showed exclusively negative SHAP values, consistently reducing ISS probability with elevated symptoms (red) clustering more densely on the left, suggesting higher levels of internalizing behavior were associated with decreased ISS risk. In contrast, *disruptive behavior* and *emotion problems* (referring to emotional regulation challenges) were associated with increased ISS likelihood. Cohort features (*yr1_k*) and starting the study in first grade (*yr1_gr1*) showed narrow SHAP distributions near zero, indicating minimal predictive influence.

### Top Predictors of Out of School Suspension 

Figure 2 shows the top 10 predictors of OSS. *Family involvement*, *internalizing problems*, and *academic performance* emerged as the most influential, all showing negative SHAP values indicative of a protective effect with higher values reducing predicted OSS probability. Most remaining predictors showed SHAP values on both sides of the axis, indicating no distinct directional relationship with OSS; exceptions were *prosocial behavior*, which served as a protective factor, and *disruptive behavior*, which emerged as a risk factor. Gender (*female*) and *cohort* showed minimal influence, with SHAP values clustered near zero.

### Top Predictors of Arrest

Figure 3 shows the top 10 predictors of arrest. *Family problems*, *prosocial behavior*, and *family involvement* showed the largest overall contributions. All three had negative SHAP values, indicating protective effects. Lower values in *family problems,* shown by blue dots clustered on the left, indicate a low likelihood of arrest. Higher values in *prosocial behavior* and *family involvement*—indicated by red dots also on the left side—were each linked to reduced arrest risk. Poorer *academic performance* and higher *disruptive behavior* were associated with increased risk, while higher *internalizing* and *concentration problems* were associated with negative SHAP values, suggesting that higher levels of these symptoms are interpreted by the model as decreasing likelihood. Gender (*female*), *cohort*, and condition (*group*) showed modest, symmetric SHAP distributions, indicating limited differential impact.

### Comparing the Strength of Predictors in the RCT Conditions

The relative strength of predictors was highly consistent across the Tier 1 only and Tier 1 + 2 conditions across models. As shown in Supplementary Information Figures S2, S3, and S4, the differences in mean SHAP values between conditions were marginal when considering models predicting ISS, OSS, and arrest. The ranking of predictors, from most to least influential, was nearly identical in both conditions in all models, indicating that the intervention context did not meaningfully alter the predictive strength or ordering of key features in predictive models. Risk factors were interpreted similarly across both Tier 1 and Tier 1 + 2 settings.

### HLM Results

Unconditional models revealed that 14.5%, 7.6%, and 6.5% of variance in secondary ISS, OSS, and arrest, respectively, was attributable to elementary school (Tables S3–S5). HLM results are presented in Table 3; all top 10 SHAP-identified predictors were significant for each outcome. Baseline *disruptive behavior* was the strongest predictor of ISS (*B* = 0.54; *p* <.001; OR = 1.72) and OSS (*B* = 0.65; *p* <.001; OR = 1.91). For arrest, *cohort* was the strongest predictor (*B* =  − 0.77; *p* <.001; OR = 0.46), followed by *disruptive behavior* (*B* = 0.32; *p* <.001; OR = 1.37). Results were similar across intervention and comparison conditions (Tables S6–S7).

**Table 3:** Results for multilevel models examining the impact of top predictor variables identified by the SHAP analysis

	In-school suspension			Out-of-school suspension			Arrest		
	*B*	*(SE)*	*OR*	*B*	*(SE)*	*OR*	*B*	*(SE)*	*OR*
Fixed effects									
Intercept	− 1.68^***^	(0.13)	0.19	− 0.92^***^	(0.07)	0.40	− 1.19^***^	(0.09)	0.30
Level 1 (student)									
Academic performance (c)	− 0.18^***^	(0.02)	0.83	− 0.23^***^	(0.02)	0.79	− 0.08^***^	(0.02)	0.92
Concentration problems (c)	X			X			0.08^**^	(0.03)	1.08
Disruptive behavior (c)	0.54^***^	(0.05)	1.72	0.65^***^	(0.04)	1.91	0.32^***^	(0.04)	1.37
Emotion problem (c)	0.06	(0.04)	1.06	0.15^***^	(0.03)	1.16	X		
Family involvement (c)	− 0.06^**^	(0.02)	0.95	− 0.09^***^	(0.02)	0.91	− 0.09^***^	(0.02)	0.91
Family problem (c)	X			0.15^***^	(0.03)	1.16	0.23^***^	(0.04)	1.26
Free/reduced-priced lunch	X			0.53^***^	(0.04)	1.71	X		
Female	− 0.48^***^	(0.05)	0.62	− 0.47^***^	(0.04)	0.63	− 0.29^***^	(0.04)	0.75
Grade in year 1: K	− 0.90^***^	(0.08)	0.41	X			X		
Grade in year 1: 1	− 0.54^***^	(0.07)	0.58	X			X		
Internalizing problem (c)	− 0.36^***^	(0.04)	0.70	− 0.32^***^	(0.03)	0.73	− 0.12^***^	(0.03)	0.89
Prosocial behavior (c)	− 0.11^**^	(0.04)	0.90	0.09^**^	(0.03)	1.10	0.07^*^	(0.03)	1.07
Level 2 (year 1 school)									
Cohort	− 0.92^***^	(0.25)	0.40	− 0.58^***^	(0.12)	0.56	− 0.77^***^	(0.13)	0.46
Intervention Condition	X			X			0.09	(0.11)	1.10
Random parameters									
Level 2: year 1 school									
Var (cons)	0.464	(0.681)		0.105	(0.324)		0.119	(0.344)	

*OR* odds ratio, *X* variable not included because only top 10 predictors for each outcome were included., *p <.05, **p <.01, ***p <.001

## Discussion

The current study used ML methodology and RCT data to identify elementary-level predictors of long-term non-response to MTSS-B, measured as ISS, OSS, and arrest in grades 6–12. Final algorithms identified the top 10 predictors for each outcome, though not all provided clear and meaningful relationships. Notably, family involvement emerged as a top-three predictor across all three outcomes, and internalizing problems ranked in the top three for both suspension outcomes; gender was uniquely prominent for ISS and academic performance for OSS. Predictor strength was similar across Tier 1 and Tier 1 + 2 conditions. Combining ML with HLM demonstrated how these approaches can complement one another by using ML for predictor selection and HLM to estimate relative predictor-outcome relationships. These findings help to identify potential risk factors that may be useful screening tools within tiered supports, allowing for early intervention rather than waiting for non-response to occur.

### Differences Between the Tier 1 + 2 and Tier 1 Only Groups

Predictor strength and ordering were largely consistent across Tier 1 and Tier 1 + 2 conditions, indicating that baseline predictors of ISS, OSS, and arrest are similar regardless of intervention context. For example, family problems were a top predictor of arrest in both conditions, suggesting they do not differentially predict non-response by group. Importantly, this does not speak to whether Tier 1 + 2 access reduced the overall likelihood of experiencing these outcomes. Instead, our results indicate that students who did experience them had similar baseline characteristics across conditions, suggesting the availability of Tier 2 supports did not uniquely address the needs of students with a specific baseline risk profile. It remains possible, for instance, that Tier 2 access reduced suspension or arrest risk broadly by institutionalizing more restorative responses to misbehavior, rather than by targeting a specific risk factor.

### Implications for Prevention

Family involvement emerged as a top protective factor across all models, consistent with research linking parental engagement to reduced youth violence, improved academic achievement, and decreased juvenile justice involvement (Chen & Gregory, 2009; Dong & Wiebe, 2018). From an ecological perspective, this is particularly notable given that parental involvement tends to increase most at the Tier 3 level (Garbacz et al., 2018), suggesting an opportunity for earlier engagement. School-led efforts to promote family involvement alongside behavior-focused interventions may reduce risk for exclusionary discipline and justice involvement. Parent engagement interventions warrant consideration as a Tier 2 support for students showing early risk indicators (Garbacz et al., 2018), and ideally, family engagement should begin prior to the emergence of need for Tier 2 or 3 interventions.

Notably, the best-fitting algorithms for predicting each of our outcomes of interest differed from one another. For example, family problems ranked among the top predictors for arrest but not for ISS, whereas family involvement at school did appear in the ISS model. This suggests that screening tools should be tailored to the specific outcome of interest, as predictors effective for one outcome may not translate to another.

### Limitations and Future Directions

While ML is not a traditional strategy for examining intervention outcomes, it offers a novel approach to identifying non-response predictors. Importantly, ML results cannot be interpreted causally or like traditional regression coefficients. Rather than reflecting mean outcome changes per unit increase in a predictor, ML results reflect how the algorithm uses variables to generate predictions. Additionally, ML cannot yet account for nested data structures—an important limitation in educational contexts where students are nested within classrooms, schools, and districts. The inclusion of HLM addressed this limitation, providing estimates of mean predictor impacts and relative strength of statistical associations. However, our HLM analysis was limited in that we only nested students in their baseline school; we did not account for the multiple schools attended over time or the schools in which the students experienced the 6th through 12th grade outcomes.

The analysis presented here also has strengths, including the computing power of ML and the ability to compare multiple models. ML allowed us to include a large number of variables (Obermeyer & Emanuel, 2016). We also evaluated multiple models, including those capturing non-linear and higher-order associations (Leist et al., 2022). Practically, this approach allowed us to begin with outcomes of interest and work backwards to identify strong predictors for those outcomes.

Due to the school-level design and systems focus of the MTSS-B preventive intervention, we were only able to track availability of those programs at the school level and did not have data on which specific students received the Tier 2 interventions. Finally, we took an “ever/never” approach to dichotomizing the suspension and arrest outcomes, rather than examining time to event or specific types of events (e.g., suspensions or arrests for different offenses). This in part was due to the relatively low base rate of events, and the number of models run. Future analyses could explore specific types of offenses in greater detail.

## Conclusion

Across all three outcomes, family-related factors emerged as cross-cutting predictors: family involvement was a top predictor of ISS, OSS, and arrest, while family problems were particularly salient for arrest. Internalizing problems were prominent for both suspension outcomes, with gender uniquely predictive of ISS and academic performance of OSS. Notably, predictive algorithms differed by outcome, indicating the importance of outcome-specific screening tools. The centrality of family factors across outcomes suggests that parental engagement prior to the Tier 3 level at which families are typically involved may be a promising target for future intervention efforts (Garbacz et al., 2018). These findings may also inform the development of screening tools to help school personnel identify which students need which supports before negative outcomes occur.

## Supplementary Information

Below is the link to the electronic supplementary material.

ESM 1 Supplementary Material 1 (DOCX 3.86 MB)

## Data Availability

The outcome data used in this study are available from the Maryland Longitudinal Data System (MLDS) Center. Restrictions apply to the availability of these data. Access to restricted used data is available with permission from the MLDS Center.
