# Supplementary material for: Using Machine Learning Methodology to Identify Predictors of Non-Response to MTSS-B: a Focus on Discipline Problems and Juvenile Justice Involvement
Source: Prev Sci. 2026 May 22;27(7):1140–52. doi: 10.1007/s11121-026-01932-0 (PMC13550068; doi:10.1007/s11121-026-01932-0)
Supplement: Supplementary file 1 [file 11121_2026_1932_MOESM1_ESM.docx]

**Supplementary Information**

Figure S1

*CONSORT Diagram.*

Figure S2

*In-School Suspension (ISS) SHAP Bar Plot for the Comparison and Intervention Groups.*

Figure S3

*Out-of-School Suspension (OSS) SHAP Bar Plot for the Comparison and Intervention Groups.*

Figure S4

*Arrest SHAP Bar Plot for the Comparison and Intervention Groups.*

Table S1

*Racial/Ethnic Breakdown of the Sample (N = 16,907)*

| **Race/Ethnicity** | **Definition** | **N (%)** |
| --- | --- | --- |
| African American | Student identifies as African American. | 8357 (49.4%) |
| American Indian/Alaska Native | Student identifies as American Indian or Alaska Native. | 84 (0.5%) |
| Asian/Pacific Islander | Student identifies as Asian or Pacific Islander. | 522 (3.1%) |
| Hispanic | Student identifies as Hispanic. | 1507 (8.9%) |
| Multi-racial | Student identifies as multi-racial. | 704 (4.2%) |
| White | Student identifies as White. | 5641 (33.4%) |
| Not Available | Race/ethnicity not available. | 92 (0.5%) |

***Note.*** Although the full racial/ethnic breakdown of the sample is presented in this supplemental table, the machine learning analysis reported in the main text used a binary variable (Minority; 0 = white, all other racial/ethnic categories = 1) derived from that breakdown as a predictor rather than specific racial/ethnic categories. This decision was made because the analytic approach required binary variables to rank predictors from strongest to weakest, and disaggregating race/ethnicity into specific categories threatened to dissolve its predictive power. We operationalized this variable as white versus nonwhite to reflect scholarship documenting how systemic racism positions whiteness as the assumed norm in U.S. schooling, affording white students protection from the discriminatory disciplinary practices disproportionately experienced by students of color. We acknowledge that collapsing racially and ethnically minoritized students into a single category inadequately captures the diverse and intersecting identities held by people of color, and that the experiences of these communities are not monolithic.

Table S2

*Results for the Top Performing Machine Learning Algorithm Predicting Suspensions and Arrests (without SMOTE)*

| **Outcome** | **Model** | **Accuracy** | **Precision** | **Recall** | **F1 Score** | **Cohen Kappa** | **MCC** | **ROC AUC** |
| --- | --- | --- | --- | --- | --- | --- | --- | --- |
| **Arrest** |  |  |  |  |  |  |  |  |
|  | random_forest | 0.7599 | 0.7417 | 0.7599 | 0.7475 | 0.2975 | 0.3025 | 0.6363 |
| **In School Suspension** | |  |  |  |  |  |  |  |
|  | lda | 0.9152 | 0.8733 | 0.9152 | 0.8846 | 0.059 | 0.0933 | 0.5179 |
| **Out of School Suspension** | |  |  |  |  |  |  |  |
|  | random_forest | 0.7599 | 0.7417 | 0.7599 | 0.7475 | 0.2975 | 0.3025 | 0.6363 |
| MCC = Matthews Correlation Coefficient | | |  |  |  |  |  |  |
| ROC AUC = The Area Under the Receiver Operating Characteristic Curve | | | | | |  |  |  |

Table S3

*Intraclass Correlations Calculated from Unconditional Multilevel Models (N=16,907, 42 schools*)

|  | In-School Suspension | | Out-of-School Suspension | | Arrest | |
| --- | --- | --- | --- | --- | --- | --- |
|  | *Variance* | *(SD)* | *Variance* | *(SD)* | *Variance* | *(SD)* |
|  |  |  |  |  |  |  |
| Level 2 (Year 1 School) | 0.556 | (0.746) | 0.271 | (0.521) | 0.228 | (0.478) |
| Level 1 (Student) | 3.290 |  | 3.290 |  | 3.290 |  |
|  |  |  |  |  |  |  |
| ICC | .145 |  | .076 |  | .065 |  |

*Notes.* All outcomes were binary and reflect whether student ever experienced the event in grades 6 through 12. ICC = intraclass correlation and indicates the amount of variance occurring between schools. Students were nested in their first Year 1 school. Level 1 variance was calculated assuming a logistic distribution with variance equal to 𝜋^2^/3 = 3.29 (Hedeker, 2003; Hox et al., 2018).

Table S4

*Intraclass Correlations Calculated from Unconditional Multilevel Models, Comparison Sample Only (N=9,247, 22 schools)*

|  | In-School Suspension | | Out-of-School Suspension | | Arrest | |
| --- | --- | --- | --- | --- | --- | --- |
|  | *Variance* | *(SD)* | *Variance* | *(SD)* | *Variance* | *(SD)* |
|  |  |  |  |  |  |  |
| Level 2 (Year 1 School) | 0.398 | (0.631) | 0.201 | (0.448) | 0.154 | (0.392) |
| Level 1 (Student) | 3.290 |  | 3.290 |  | 3.290 |  |
|  |  |  |  |  |  |  |
| ICC | .108 |  | .057 |  | .045 |  |

*Notes.* All outcomes were binary and reflect whether student ever experienced the event in grades 6 through 12. ICC = intraclass correlation and indicates the amount of variance occurring between schools. Students were nested in their first Year 1 school. Level 1 variance was calculated assuming a logistic distribution with variance equal to 𝜋^2^/3 = 3.29 (Hedeker, 2003; Hox et al., 2018).

Table S5

*Intraclass Correlations Calculated from Unconditional Multilevel Models, Intervention Sample Only (N=7,660, 20 schools)*

|  | In-School Suspension | | Out-of-School Suspension | | Arrest | |
| --- | --- | --- | --- | --- | --- | --- |
|  | *Variance* | *(SD)* | *Variance* | *(SD)* | *Variance* | *(SD)* |
|  |  |  |  |  |  |  |
| Level 2 (Year 1 School) | 0.732 | (0.856) | 0.351 | (0.593) | 0.310 | (0.557) |
| Level 1 (Student) | 3.290 |  | 3.290 |  | 3.290 |  |
|  |  |  |  |  |  |  |
| ICC | .182 |  | .097 |  | .086 |  |

*Notes.* All outcomes were binary and reflect whether student ever experienced the event in grades 6 through 12. ICC = intraclass correlation and indicates the amount of variance occurring between schools. Students were nested in their first Year 1 school. Level 1 variance was calculated assuming a logistic distribution with variance equal to 𝜋^2^/3 = 3.29 (Hedeker, 2003; Hox et al., 2018).

Table S6

*Results for Multilevel Models Examining the Impact of Top Predictor Variables Identified by the SHAP Analysis, Comparison Sample Only (N=9,247, 22 schools)*

|  | In-School Suspension | | | Out-of-School Suspension | | | Arrest | | |
| --- | --- | --- | --- | --- | --- | --- | --- | --- | --- |
|  | *B* | *(SE)* | *OR* | *B* | *(SE)* | *OR* | *B* | *(SE)* | *OR* |
| Fixed Effects |  |  |  |  |  |  |  |  |  |
| Intercept | -1.76^***^ | (0.15) | 0.17 | -0.86^***^ | (0.07) | 0.42 | -1.19^***^ | (0.06) | 0.31 |
| Level 1 (Student) |  |  |  |  |  |  |  |  |  |
| Academic performance (c) | -0.21^***^ | (0.03) | 0.81 | -0.24^***^ | (0.02) | 0.79 | -0.08^*^ | (0.03) | 0.92 |
| Concentration problems (c) | X |  |  | X |  |  | 0.10^*^ | (0.04) | 1.10 |
| Disruptive behavior (c) | 0.65^***^ | (0.07) | 1.91 | 0.61^***^ | (0.06) | 1.84 | 0.32^***^ | (0.05) | 1.38 |
| Emotion problem (c) | 0.06 | (0.06) | 1.06 | 0.15^***^ | (0.04) | 1.16 | X |  |  |
| Family involvement (c) | -0.04 | (0.03) | 0.96 | -0.07^***^ | (0.02) | 0.93 | -0.08^***^ | (0.02) | 0.93 |
| Family problem (c) | X |  |  | 0.20^***^ | (0.05) | 1.22 | 0.25^***^ | (0.05) | 1.29 |
| Free/reduced-price lunch | X |  |  | 0.50^***^ | (0.06) | 1.65 | X |  |  |
| Female | -0.45^***^ | (0.07) | 0.64 | -0.51^***^ | (0.05) | 0.60 | -0.33^***^ | (0.06) | 0.72 |
| Grade in year 1: K | -0.88^***^ | (0.11) | 0.41 | X |  |  | X |  |  |
| Grade in year 1: 1 | -0.50^***^ | (0.10) | 0.60 | X |  |  | X |  |  |
| Internalizing problem (c) | -0.36^***^ | (0.06) | 0.70 | -0.33^***^ | (0.04) | 0.72 | -0.09^*^ | (0.04) | 0.92 |
| Prosocial behavior (c) | -0.09 | (0.05) | 0.91 | 0.05 | (0.04) | 1.06 | 0.10^*^ | (0.05) | 1.11 |
|  |  |  |  |  |  |  |  |  |  |
| Level 2 (Year 1 School) |  |  |  |  |  |  |  |  |  |
| Cohort | -0.94^**^ | (0.31) | 0.39 | -0.70^***^ | (0.14) | 0.50 | -0.77^***^ | (0.13) | 0.46 |
| Random Parameters |  |  |  |  |  |  |  |  |  |
| Level 2: Year 1 School |  |  |  |  |  |  |  |  |  |
| Var (cons) | 0.329 | (0.574) |  | 0.060 | (0.245) |  | 0.045 | (0.212) |  |

*Notes.* All outcomes were binary and reflect whether student ever experienced the event in grades 6 through 12. Multilevel logistic modeling was used for all outcomes. Students were nested in their first Year 1 school. (c) indicates the variable was grand mean centered. X indicates the variable was not included in the model. ^***^ *p* < .001; ^**^ *p* < .01; ^*^ *p* < .05.

Table S7

*Results for Multilevel Models Examining the Impact of Top Predictor Variables Identified by the SHAP Analysis, Intervention Sample Only (N=7,660, 20 schools)*

|  | In-School Suspension | | | Out-of-School Suspension | | | Arrest | | |
| --- | --- | --- | --- | --- | --- | --- | --- | --- | --- |
|  | *B* | *(SE)* | *OR* | *B* | *(SE)* | *OR* | *B* | *(SE)* | *OR* |
| Fixed Effects |  |  |  |  |  |  |  |  |  |
| Intercept | -1.59^***^ | (0.22) | 0.20 | -1.00^***^ | (0.12) | 0.37 | -1.10^***^ | (0.13) | 0.33 |
| Level 1 (Student) |  |  |  |  |  |  |  |  |  |
| Academic performance (c) | -0.14^***^ | (0.04) | 0.87 | -0.23^***^ | (0.03) | 0.80 | -0.07^*^ | (0.04) | 0.93 |
| Concentration problems (c) | X |  |  | X |  |  | 0.06 | (0.04) | 1.06 |
| Disruptive behavior (c) | 0.42^***^ | (0.07) | 1.53 | 0.70^***^ | (0.06) | 2.01 | 0.32^***^ | (0.05) | 1.38 |
| Emotion problem (c) | 0.06 | (0.06) | 1.06 | 0.15^**^ | (0.05) | 1.16 | X |  |  |
| Family involvement (c) | -0.07^*^ | (0.03) | 0.93 | -0.11^***^ | (0.02) | 0.89 | -0.12^***^ | (0.02) | 0.89 |
| Family problem (c) | X |  |  | 0.09 | (0.05) | 1.09 | 0.21^***^ | (0.05) | 1.24 |
| Free/reduced-price lunch | X |  |  | 0.57^***^ | (0.06) | 1.77 | X |  |  |
| Female | -0.51^***^ | (0.08) | 0.60 | -0.42^***^ | (0.06) | 0.66 | -0.23^***^ | (0.06) | 0.79 |
| Grade in year 1: K | -0.93^***^ | (0.12) | 0.39 | X |  |  | X |  |  |
| Grade in year 1: 1 | -0.59^***^ | (0.11) | 0.55 | X |  |  | X |  |  |
| Internalizing problem (c) | -0.36^***^ | (0.06) | 0.69 | -0.31^***^ | (0.04) | 0.73 | -0.15^***^ | (0.04) | 0.86 |
| Prosocial behavior (c) | -0.12^*^ | (0.06) | 0.88 | 0.14^**^ | (0.04) | 1.15 | 0.04 | (0.05) | 1.04 |
|  |  |  |  |  |  |  |  |  |  |
| Level 2 (Year 1 School) |  |  |  |  |  |  |  |  |  |
| Cohort | -0.90^*^ | (0.40) | 0.41 | -0.46^*^ | (0.20) | 0.63 | -0.77^***^ | (0.23) | 0.46 |
| Random Parameters |  |  |  |  |  |  |  |  |  |
| Level 2: Year 1 School |  |  |  |  |  |  |  |  |  |
| Var (cons) | 0.625 | (0.790) |  | 0.150 | (0.387) |  | 0.202 | (0.450) |  |

*Notes.* All outcomes were binary and reflect whether student ever experienced the event in grades 6 through 12. Multilevel logistic modeling was used for all outcomes. Students were nested in their first Year 1 school. (c) indicates the variable was grand mean centered. X indicates the variable was not included in the model. ^***^ *p* < .001; ^**^ *p* < .01; ^*^ *p* < .05
